# The Impact of Ischaemic Stroke Subtype on 30-day Hospital Readmissions

**DOI:** 10.1155/2018/7195369

**Published:** 2018-12-03

**Authors:** Anna Therese Bjerkreim, Andrej Netland Khanevski, Henriette Aurora Selvik, Ulrike Waje-Andreassen, Lars Thomassen, Halvor Naess, Nicola Logallo

**Affiliations:** ^1^Department of Clinical Medicine, University of Bergen, Norway; ^2^Department of Neurology, Haukeland University Hospital, Norway; ^3^Norwegian Health Association, Oslo, Norway; ^4^Centre for Age-Related Medicine, Stavanger University Hospital, Norway; ^5^Department of Neurosurgery, Haukeland University Hospital, Norway

## Abstract

**Background:**

Stroke aetiology may affect the risk and causes of readmission after ischaemic stroke (IS) and transient ischaemic attack (TIA) due to differences in risk factors, functional outcome, and treatment. We aimed to examine frequencies, causes, and risk of 30-day readmission by stroke subtype, determine predictors of 30-day readmission, and study the impact of 30-day readmissions on one-year mortality.

**Methods:**

All surviving patients admitted with IS or TIA from July 2007 to December 2013 were followed by review of medical records for all unplanned readmissions within 30 days after discharge. Stroke subtype was classified as large-artery atherosclerosis (LAA), cardioembolism (CE), small vessel occlusion (SVO), stroke of other determined aetiology (SOE), or stroke of undetermined aetiology (SUE). Cox regression analyses were performed to assess the risk of 30-day readmission for the stroke subtypes and identify predictors of 30-day readmission, and its impact on one-year mortality.

**Results:**

Of 1874 patients, 200 (10.7%) were readmitted within 30 days [LAA 42/244 (17.2%), CE 75/605 (12.4%), SVO 12/205 (5.9%), SOE 6/32 (18.8%), SUE 65/788 (8.3%)]. The most frequent causes of readmissions were stroke-related event, infection, recurrent stroke/ TIA, and cardiac disease. After adjusting for age, sex, functional outcome, length of stay, and the risk factor burden, patients with LAA and SOE subtype had significantly higher risks of readmission for any cause, recurrent stroke or TIA, and stroke-related events. Predictors of 30-day readmission were higher age, peripheral arterial disease, enteral feeding, and LAA subtype. Thirty-day readmission was an independent predictor of one-year mortality.

**Conclusions:**

Patients with LAA or SOE have a high risk of 30-day readmission, possibly caused by an increased risk of recurrent stroke and stroke-related events. Awareness of the risk of readmission for different causes and appropriate handling according to stroke subtype may be useful for preventing some readmissions after stroke.

## 1. Introduction

Stroke treatment has improved considerably over the last decades, but 30-day readmission rates after stroke have remained unchanged in Norway [1]. Thirty-day readmission rates range from 6.0% to 15.0%, with infections, recurrent stroke, and cardiac conditions as leading causes [1–8]. Reducing 30-day readmission is an important target in reducing post-stroke morbidity, mortality, and health care costs [9]. Many studies have attempted to identify predictors of readmission after stroke as this might help detect high-risk patients, but results across studies are inconsistent [2]. Factors previously associated with 30-day readmission include higher age, functional status, prior stroke or cardiac disease, longer hospitalisation, and complications during stroke admission [2–4].

Aetiologies causing ischaemic stroke (IS) and transient ischaemic attack (TIA) are categorised into different stroke subtypes [10]. Cardiovascular risk factors, neurological deficits, functional outcome, and early recurrence differ between the subtypes [11–13]; therefore, stroke subtype may influence the rates and causes of 30-day readmission. In lack of existing prediction models on the risk of 30-day readmission after stroke, estimating the risk of readmission for the different stroke subtypes might be a valuable approach for detecting patients at higher risk of readmission. This would also be easily accessible in a clinical setting. However, the impact of stroke subtype on 30-day readmission risk is not clear.

The aims of our study were to examine (1) frequency, causes, and risk of 30-day readmission for the stroke subtypes, (2) factors associated with unplanned readmission within 30 days, and (3) the impact of 30-day readmission on one-year mortality.

## 2. Methods

All patients with IS or TIA admitted to the Stroke Unit at the Department of Neurology, Haukeland University Hospital, from July 2007 to December 2013, were prospectively registered in the Bergen NORSTROKE Registry. The Stroke Unit serves a well-defined geographical area with approximately 275,000 inhabitants. Patients with residence outside the geographic area of the hospital at the time of index hospitalisation were excluded from the study to ensure complete follow-up after discharge.

IS was defined as an episode of neurologic deficit lasting >24 hours because of ischaemic lesions or transient ischaemic attacks where computed tomography or magnetic resonance imaging showed infarctions related to the clinical findings [14]. TIA was a clinical diagnosis defined as transient focal cerebral dysfunction lasting < 24 hours with no objective evidence of brain infarction on imaging. Electrocardiogram, echocardiography, and duplex ultrasound of the carotid arteries were obtained during hospital admission. Stroke aetiology was classified by a stroke neurologist (HN) according to the Trial of Org 10172 in Acute Stroke Treatment (TOAST) criteria as large-artery atherosclerosis (LAA), cardioembolism (CE), small vessel occlusion (SVO), stroke of other determined aetiology (SOE), or stroke of undetermined aetiology (SUE) [10]. The National Institutes of Health Stroke Scale (NIHSS) was used to assess stroke severity on admission and day 7, or at discharge if earlier. Short-term functional outcome was determined by modified Rankin Scale (mRS) and Barthel Index (BI) on day 7 or at discharge if earlier. Clinical characteristics, treatment, medical history, comorbidity, complications, and the discharge destination were registered before discharge from the Stroke Unit. The number of traditional cardiovascular risk factors was defined as the risk factor burden (0, 1, 2, or ≥ 3). These included hypertension, diabetes mellitus, smoking, angina pectoris, peripheral arterial disease, and prior myocardial infarction. Secondary prevention from the day of discharge was based on the Norwegian guidelines for stroke treatment [15].

Two clinicians (ATB & ANK) collected information on readmission and death by reviewing electronic medical records from all ten hospitals within the region of the Western Norway Regional Health Authorities. Readmission was defined as an unplanned admission to any department at any of the hospitals within the region of the Western Norway Regional Health Authorities. All unplanned readmissions within 30 days after discharge were registered and divided into predefined categories (any cause, recurrent stroke, stroke-related event, seizure, infection, cardiac disease, fracture, venous thromboembolism, gastrointestinal haemorrhage, and other cause). Only the first readmission within each category was registered for each patient. Patients who died during index hospitalisation and patients discharged to palliative care were excluded from the analyses.

The study was approved by the Western Regional Ethics Committee, and written informed consent was obtained from all patients or their legal guardian.

Statistical analyses were performed using Stata 14.0 (Stata Corporation, College Station, TX, USA). Baseline characteristics were assessed by chi-squared test, Student's* t*-test, Mann–Whitney's* U*-test, or Kruskal-Wallis* H*-test as appropriate. The risks of readmission for any cause and for the most common categories of readmission were assessed by Cox regression analyses for each subtype compared to patients with other stroke subtypes. In addition to the stroke subtype, all analyses included age, sex, mRS score, length of stay, and the risk factor burden to adjust for potential confounding factors. Predictors of the 30-day readmission were assessed by Cox regression with stepwise backward elimination of all significant parameters (*P*<.05) from the univariate analyses. Age, sex, mRS score, and length of stay were forced into the multivariate analyses to adjust for potential confounding factors. The impact of 30-day readmission on one-year mortality was studied by Cox regression analyses adjusted for age, sex, mRS score, and risk factors for cerebrovascular disease and death, after excluding patients who died within 30 days after discharge.

## 3. Results

A total of 1989 patients were admitted to the stroke unit and diagnosed with IS (89.6%) or TIA (10.4%), of whom 101 (5.1%) died in the stroke unit, and 14 (0.7%) were discharged to palliative care. The final study population consisted of 1874 patients, of whom 200 (10.7%) were readmitted within 30 days (10.9% of IS patients, 9.2% of TIA patients, p = 0.475). The distribution of readmissions among the stroke subtypes was as follows: LAA 42/244 (17.2%), CE 75/605 (12.4%), SVO 12/205 (5.9%), SOE 6/32 (18.8%), and SUE 65/788 (8.3%). Nineteen patients had two unplanned readmissions within 30 days, and two patients had three unplanned readmissions within 30 days. Median time to first unplanned readmission was 11 days (IQR 5-19).

Baseline characteristics of the study population by 30-day readmission status are presented in Table 1. Patients who were readmitted within 30 days were in general older, had a poorer short-term functional outcome (higher mRS score and lower BI score), and a higher NIHSS score at discharge than patients not readmitted within 30 days. Readmitted patients also had more cardiovascular comorbidity and a higher risk factor burden, and more frequently experienced complications during the index hospitalisation than patients not readmitted within 30 days. Patients with LAA subtype were more likely to be readmitted, whereas patients with SVO or SUE subtype were less likely to be readmitted.

The most frequent cause of readmission was stroke-related events: 10 with evolving strokes (NIHSS score worsening ≤ 4 points from NIHSS score at discharge with symptoms from the same vascular territory as the index stroke), 19 with aggravation of existing neurological deficits (neurological symptoms not measurable by the NIHSS score), 2 with haemorrhagic transformation, 3 with neurological complications after carotid endarterectomy, 1 with extracranial haemorrhage, 1 with carotid dissection, and 15 with other neurological symptoms. The length of index hospitalisation was slightly shorter for patients that were readmitted with a stroke-related event compared to patients that were not readmitted with a stroke-related event (median 5 days (IQR 5-8) versus median 6 days (IQR 3-10), p=0.193). Infection was the second most frequent cause of readmission, followed by recurrent stroke or TIA, and cardiac disease. The causes of readmission were unevenly distributed among the stroke subtypes (Table 2).

Table 3 shows the distribution of age, sex, mRS score, length of stay, and the risk factor burden among the stroke subtypes. After adjusting for these factors, patients with stroke due to LAA had an increased risk of readmission for any cause (HR=1.76, 95% CI 1.22-2.53, p=0.002), readmission with recurrent stroke or TIA (HR = 3.31, 95% CI 1.55-7.07, p=0.002), and readmission with stroke-related events (HR=2.98, 95% CI 1.55-5.74, p=0.001). Patients with SOE had an increased risk of readmission for any cause (HR=2.81, 95% CI 1.11-7.10, p=0.029), recurrent stroke (HR=6.00, 95% CI 1.26-28.57, p=0.024), and stroke-related events (HR=4.27, 95% CI 1.19-15.35, p=0.026). The recurrent strokes in the SOE group were ascribed to dissection of the middle cerebral artery, giant cerebral aneurysm, and pancreatic cancer, respectively. Patients with SUE had a lower risk of readmission for any cause compared to other subtypes (HR=0.71, 95% CI 0.52-0.97, p=0.031). Patients with CE subtype had an increased risk of readmission due to cardiac disease (HR=2.56, 95% CI 1.07-6.09, p=0.034). Exclusion of SOE from the regression models did not alter any findings.

Factors independently associated with 30-day readmission from the final Cox regression model were increasing age, history of peripheral arterial disease, enteral feeding during index hospitalisation (nasogastric tube or percutaneous endoscopic gastrostomy), and LAA subtype (Table 4).

Within 30 days, 61 (3.3%) patients died, of whom 29 (47.5%) had been readmitted. There were no significant differences in 30-day mortality between stroke subtypes (LAA 3.3%, CE 4.1%, SVO 1%, SOE 0%, and SUE 3.3%, p = 0.203). Within one year, 139 (7.4%) additional patients died, of whom 36 (25.9%) had been readmitted within 30 days. One-year mortality significantly differed between the stroke subtypes (LAA 11.8%, CE 14.0%, SVO 3.4%, SOE 3.1%, and SUE 10.2%, p = 0.001). Thirty-day readmission significantly increased the risk of one-year mortality in patients surviving 30 days after discharge after adjusting for age, sex, mRS score, and risk factors for cerebrovascular disease and death (HR 2.57, 95% CI 1.75 – 3.78, p < 0.001).

## 4. Discussion

This is one of the first and the current largest studies investigating the impact of stroke subtype on 30-day readmission after ischaemic stroke or TIA. Our study demonstrates that 30-day readmission varies depending on the stroke subtype, ranging from 5.9% for patients with SVO to 17.2% for patients with LAA and 18.8% for patients with SOE. A study performed in a younger stroke population found that 14% of patients with thrombotic strokes were readmitted within 30 days, whereas 22% of patients with CE strokes were readmitted within 30 days [16]. Another study found that 22% of patients with CE and 20% of patients with LAA subtype were readmitted within 30 days, whereas SUE had the lowest rate of readmission, with 8% readmitted within 30 days [4]. These discrepancies are likely due to study populations with different demography, lower mean ages, and different distribution of stroke subtypes than ours.

The varying readmission rates between the stroke subtypes could be caused by differences in age, functional outcome, and risk factors. After adjusting for these factors, patients with SVO, which had the lowest number of readmission, did not have a lower risk of readmission compared to other subtypes. However, the risk of 30-day readmission was significantly increased in patients with LAA and SOE subtype, whereas patients with SUE had a significantly lower risk of readmission after adjusting for confounders.

Two of the most common causes of readmission were stroke-related events and recurrent stroke. The risk of recurrent stroke is highest in the early phase after the initial IS or TIA [11]. Awareness and attention towards recurrent strokes and a low threshold for readmission in cases which could represent recurrent strokes, may contribute to a high rate of readmissions with stroke-related events such as aggravation or fluctuation of existing neurologic symptoms. Thus, education of patients and their families in the natural course of the post-stroke period may reduce some of these readmissions.

In concordance with recent studies on 30-day recurrence, only 1.8 % were readmitted with a recurrent stroke within 30 days [17, 18]. However, 4.5% of patients with LAA subtype were readmitted with recurrent stroke within 30 days. LAA subtype has previously been associated with early recurrent stroke [11, 12]. This could be caused by repetitive shedding of thrombi from unstable atherosclerotic plaques triggered by shear stress [19], or cerebral ischaemia induced by carotid surgery in some patients, but the reason for the high risk of early recurrence in patients with LAA is not yet fully understood. Patients with LAA or SOE subtype had increased risks of recurrent stroke and stroke-related events, which likely explains their overall increased risk of 30-day readmission. LAA subtype was also an independent predictor of 30-day readmission, probably due to increased recurrence of recurrent stroke or TIA and aggravation of neurological symptoms. Few studies have reported stroke recurrence for patients with SOE as a result of low numbers of subjects. The increased risk of recurrence as well as readmission for any cause and stroke-related events for patients with SOE in our study could be a chance finding due to a low number of subjects and should be interpreted with caution. However, it is possible that SOE includes some rare aetiologies with an especially high risk of early recurrence.

Infection was a frequent complication during index hospitalisation in our study, and the second most common cause of readmission. Early infections after stroke are associated with enteral feeding which might explain why enteral feeding independently increased the risk of 30-day readmission [20]. Poor functional outcome has also been associated with post-stroke infections [21]. Even though there were differences in short-term functional outcome between the stroke subtypes, the median mRS score was 2 or less for all stroke subtypes. This might explain why we found no differences in infection-related readmission between the stroke subtypes. Poor functional outcome and length of stay have been associated with early readmission [2, 3]. This could be due to an increased risk of infection in patients with poor functional outcome as well as a higher risk of undetected medical complications if patients are discharged too early [21]. The present study unexpectedly found that poor functional outcome and length of stay were not associated with readmission. This could be due to higher rates of cardiovascular and stroke-related readmissions than infections, which, in contrast to infections, likely are not affected by functional outcome or length of stay to the same extent. This might also explain why we found no differences in readmission between IS and TIA patients.

Thirty-day readmission was significantly associated with an increased risk of one-year mortality. This has previously been demonstrated [9] and could perhaps be due to interference with functional recovery after stroke if the patient is readmitted. Also, the cause of readmission might contribute to a worse prognosis and higher post-stroke mortality, as demonstrated for post-stroke pneumonia and recurrent stroke [22, 23]. Even though most readmissions are unavoidable when reviewed retrospectively [24], a reduction in readmission rates have been demonstrated with early outpatient visits by physicians, and with education of patients about relevant diagnoses, and confirmation of the medication plan [25–27]. Sufficient monitoring and care for prevention of medical complications, both during hospitalisation and in the immediate post-hospital care setting, may be key factors in preventing some readmissions. In addition, a thorough evaluation for detecting the underlying cause of the stroke and awareness of readmission risk and treatment according to the stroke subtype may be useful in preventing some readmissions after stroke.

A weakness of the present study is the lack of information in the few cases where patients were transferred to other hospital departments. It is unlikely that we have missed some readmissions, as we exclusively included patients served by our hospital and obtained data on readmissions from all ten hospitals in our region. Our study is unable to demonstrate a direct, causative relationship between stroke subtype and readmissions as it was an observational study. The study-design limits the generalisability due to possible selection bias, but it provides precise and verified clinical data with the use of a comprehensive registry and medical record review, contributing to a better understanding of underlying causes of the readmissions.

## 5. Conclusions

Readmission within 30 days after IS and TIA remains frequent in Norway. The risk and causes of 30-day readmission differed between the stroke subtypes and were especially high in patients with LAA and SOE. Stroke subtype may substitute as a 30-day readmission risk estimator in lack of more precise risk-estimation models, but more studies are needed to confirm our findings.

## Figures and Tables

**Table 1:** Baseline Characteristics of the Study Population Stratified by 30-day Readmission.

	Readmitted N = 200	Not readmitted N = 1674	P
Age (years), mean ± SD	77.2 ± 11.8	72.8 ± 13.7	<0.001
Male sex	105 (52.5)	931 (55.6)	0.402
Stroke severity and functional outcome			
mRS score at discharge, median (IQR)	2 (1, 4)	2 (1, 3)	<0.001
NIHSS score at discharge, median (IQR)	3 (1, 8)	1 (0, 4)	<0.001
BI score at discharge, median (IQR)	90 (40, 100)	100 (75, 100)	<0.001
Stroke subtype			
Atherosclerosis	42 (21.0)	202 (12.1)	<0.001
Cardioembolism	75 (37.5)	530 (31.7)	0.095
Small vessel occlusion	12 (6.0)	193 (11.5)	0.018
Other determined aetiology	6 (3.0)	26 (1.6)	0.136
Undetermined aetiology	65 (32.5)	723 (43.2)	0.004
Comorbidities			
Prior stroke	49 (24.5)	324 (19.4)	0.085
Peripheral arterial disease	26 (13.0)	110 (6.6)	0.001
Diabetes	39 (19.5)	244 (14.6)	0.066
Angina pectoris	40 (20.0)	220 (13.1)	0.008
Myocardial infarction	35 (17.5)	235 (14.0)	0.188
Hypertension	133 (66.5)	922 (55.1)	0.002
Atrial fibrillation	70 (35.0)	475 (28.4)	0.051
Prior/current smoking	122 (61.0)	965 (57.7)	0.364
Risk factor burden			0.007
0	44 (22.0)	463 (27.7)	
1	66 (33.0)	643 (38.4)	
2	53 (26.5)	377 (22.5)	
≥3	37 (18.5)	191 (11.4)	
Treatment			
Intravenous thrombolysis	33 (16.5)	274 (16.4)	0.962
Thrombectomy	6 (3.0)	22 (1.3)	0.063
Complications during the stroke hospitalisation			
Urinary tract infection	38 (19.0)	187 (11.2)	0.001
Incontinence	31 (15.5)	211 (12.6)	0.248
Urinary retention	55 (27.5)	325 (19.4)	0.007
Pneumonia	24 (12.0)	105 (6.3)	0.002
Enteral feeding	29 (14.5)	106 (6.3)	<0.001
Seizures	9 (4.5)	36 (2.2)	0.040
Stroke in progression	23 (11.5)	147 (8.8)	0.206
Any complication	100 (50.0)	637 (38.1)	0.001
Length of stay, median (IQR)	6 (3, 11)	6 (3, 10)	0.553
Discharged destination			
Home	77 (38.5)	928 (56.0)	<0.001
Home nursing	28 (14.0)	166 (10.0)	0.081
Rehabilitation department	10 (5.0)	141 (8.5)	0.087
Nursing home	69 (34.5)	376 (22.7)	<0.001
Other department	16 (8.0)	47 (2.8)	<0.001

SD: standard deviation, mRS: modified Rankin Scale, IQR: interquartile range, NIHSS: National Institutes of Health Stroke Scale, BI: Barthel Index.

Data are expressed as n (%) unless specified.

**Table 2:** Causes of unplanned readmissions within 30 days after IS or TIA by stroke subtype.

Cause of readmission	All Stroke n=1874	LAA n=244	CE n=605	SVO n=205	SOE n=32	SUE n=788	P
**Any cause**	**200 (10.7)**	**42 (17.2)**	**75 (12.4)**	**12 (5.9)**	**6 (18.8)**	**65 (8.3)**	**<0.001**
**Recurrent stroke**	**34 (1.8)**	**11 (4.5)**	**8 (1.3)**	**1 (0.5)**	**3 (9.4)**	**11 (1.4)**	**<0.001**
IS	26 (1.4)	6 (2.5)	7 (1.2)	1 (0.5)	3 (9.4)	9 (1.1)	0.001
TIA	4 (0.2)	4 (1.6)	0	0	0	0	<0.001
ICH	4 (0.2)	1 (0.4)	1 (0.2)	0	0	2 (0.3)	0.898
**Stroke-related event**	**51 (2.7)**	**16 (6.6)**	**11 (1.8)**	**4 (2.0)**	**3 (9.4)**	**17 (2.2)**	**<0.001**
**Seizures**	**5 (0.3)**	**0**	**3 (0.5)**	**0**	**0**	**2 (0.3)**	**0.647**
**Infections**	**37 (2.0)**	**7 (2.9)**	**13 (2.2)**	**4 (2.0)**	**0**	**13 (1.7)**	**0.703**
Pneumonia	19 (1.0)	3 (1.2)	6 (1.0)	3 (1.5)	0	7 (0.9)	0.913
UTI	9 (0.5)	1 (0.4)	4 (0.7)	0	0	4 (0.5)	0.810
Sepsis	5 (0.3)	2 (0.8)	1 (0.2)	1 (0.5)	0	1 (0.1)	0.395
Other	4 (0.2)	1 (0.4)	2 (0.3)	0	0	1 (0.1)	0.806
**Cardiac disease**	**26 (1.4)**	**1 (0.4)**	**17 (2.8)**	**0**	**0**	**8 (1.0)**	**0.005**
MI	6 (0.3)	0	1 (0.2)	0	0	5 (0.6)	0.350
Arrhythmias	5 (0.3)	1 (0.4)	4 (0.7)	0	0	0	0.167
Other	16 (0.9)	0	13 (2.2)	0	0	3 (0.4)	0.001
**Fractures **	**7 (0.4)**	**0**	**5 (0.8)**	**0**	**0**	**2 (0.3)**	**0.244**
**VT**	**3 (0.2)**	**1 (0.4)**	**1 (0.2)**	**0**	**0**	**1 (0.1)**	**0.846**
**GI bleeding**	**4 (0.2)**	**0**	**2 (0.3)**	**1 (0.5)**	**0**	**1 (0.1)**	**0.739**
**Other causes**	**49 (2.6)**	**13 (5.3)**	**20 (3.3)**	**3 (1.5)**	**0**	**13 (1.7)**	**0.011**

IS: ischaemic stroke, TIA: transient ischaemic attack, LAA: large artery atherosclerosis, CE: cardioembolism, SVO: small vessel occlusion, SOE: stroke of other determined aetiology, SUE: stroke of undetermined aetiology, ICH: intracerebral haemorrhage, UTI: urinary tract infection, MI: myocardial infarction, VT: venous thromboembolism, GI: gastrointestinal.

Data are presented as n (%). Only the first readmission within each category is counted.

**Table 3:** Age, sex, modified Rankin Scale score, length of stay and risk factor burden by stroke subtype.

	LAA N=244	CE N=605	SVO N=205	SOE N=32	SUE N=788	P
Age (years), median (IQR)	75 (66, 82)	79 (68, 86)	69 (60, 80)	47 (38, 56)	74 (64, 83)	<0.001
Male sex, N (%)	159 (65.2)	318 (52.6)	123 (60.0)	21 (65.6)	415 (52.7)	0.002
mRS score, median (IQR)	2 (0, 3)	2 (1, 4)	2 (1, 3)	1 (0, 2)	1 (0, 3)	<0.001
Length of stay (days)	5 (3, 9)	6 (3, 10)	6 (3, 10)	8 (5, 12)	6 (3, 9)	0.029
Risk factor burden, N (%)						<0.001
0	34 (13.9)	168 (27.8)	55 (26.8)	15 (46.9)	235 (29.8)	
1	83 (34.0)	234 (38.7)	86 (42.0)	17 (53.1)	289 (36.7)	
2	70 (28.7)	142 (23.5)	42 (20.5)	0 (0.0)	176 (22.3)	
≥3	57 (23.4)	61 (10.1)	22 (10.7)	0 (0.0)	88 (11.2)	

IQR: interquartile range, mRS: modified Rankin Scale.

**Table 4:** Factors Associated with 30-day Unplanned Readmission after IS or TIA.

	Hazard Ratio	95% Confidence Interval	P
Age (years)	1.020	1.006 – 1.034	0.004
Sex (male)	0.936	0.690 – 1.268	0.667
mRS score	0.990	0.894 – 1.095	0.842
Length of stay (days)	0.997	0.973 – 1.022	0.846
Peripheral arterial disease	1.577	1.007 – 2.468	0.047
LAA subtype	1.735	1.201 – 2.505	0.003
Enteral feeding	1.861	1.112 – 3.114	0.018

IS: ischaemic stroke, TIA: transient ischaemic attack, mRS: modified Rankin Scale, LAA: large artery atherosclerosis.

## Data Availability

The data used to support the findings of this study are available from the corresponding author upon request.
